# NOXA exacerbates endoplasmic-reticulum-stress-induced intervertebral disc degeneration by activating apoptosis and ECM degradation

**DOI:** 10.1038/s41420-025-02539-0

**Published:** 2025-05-28

**Authors:** Zhiming Liu, Hui Lu, Xianjuan Zhang, Shuai Tang, Antao Lin, Shuo Han, Xuexiao Ma

**Affiliations:** 1 Department of Spinal Surgery, The Affiliated Hospital of Qingdao University, Qingdao, Shandong China; 2 Department of Clinical Laboratory, The Affiliated Hospital of Qingdao University, Qingdao, Shandong China

**Keywords:** RNAi, Cytokines

## Abstract

Intervertebral disc degeneration (IVDD) is a prevalent condition leading to low back pain. Endoplasmic reticulum stress (ERS) is strongly linked to IVDD progression, although the underlying mechanisms remain unclear. In this study, we investigated the effects of NOXA on ERS-induced IVDD. Primary nucleus pulposus cells (NPCs) were stimulated with Thapsigargin to mimic the ERS microenvironment in IVDD. Western blot analysis, PCR, immunofluorescence, and immunohistochemistry assay were performed to measure the expression levels of PERK, NOXA, and cell apoptosis- and extracellular-matrix-degradation-relevant proteins. JC-1 fluorescent probes, terminal deoxynucleotidyl transferase dUTP nick end labeling staining, and flow cytometry were used to measure mitochondrial function and apoptosis in NPCs under ERS conditions. Magnetic resonance imaging, Safranin O staining, alcian blue staining, and immunohistochemistry were performed to estimate the effects of NOXA knockdown on acupuncture-mediated IVDD in rats at both imaging and histological levels. The results showed that ERS induced and activated the PERK pathway during IVDD development. Mechanically, ERS induced NPC apoptosis and ECM degradation by upregulating PERK expression and activating NOXA expression. The genetic overexpression of NOXA inhibited cell proliferation and increased apoptosis, whereas its knockdown decreased MCL-1 expression and alleviated IVDD degeneration in human NPCs and rat models. NOXA plays a crucial role in the PERK/NOXA/MCL-1 axis, mediating the link between ERS and IVDD. Targeting NOXA expression may be an effective method for treating IVDD, laying the foundation for future research on molecular mechanisms and the development of new therapies.

## Introduction

Intervertebral disc degeneration (IVDD) contributes significantly to low back pain, posing a substantial burden on public health and the global economy [1]. The intervertebral disc comprises a central nucleus pulposus (NP) that is rich in water and surrounded by the annulus fibrosus (AF) and cartilaginous endplate (CEP); these components support the intervertebral disc in bearing various mechanical stresses during daily activities [2]. When NP cells (NPCs) undergo excessive apoptosis, this process leads to metabolic disorders in the extracellular matrix (ECM) of NPCs. Subsequently, these disorders disrupt the normal structure and physiological function of the intervertebral disc, resulting in IVDD [3–5].

Although the mechanism behind IVDD pathogenesis remains elusive, increasing evidence suggests that the endoplasmic reticulum (ER) is involved in metabolism homeostasis and IVDD progression [6, 7]. The ER plays an important role within the cell and is responsible for protein synthesis, folding, and transport. When misfolded or incorrectly folded proteins accumulate in the ER lumen, the cell initiates a defensive stress response known as ER stress (ERS) [8, 9]. When ERS occurs, the cell can sense the accumulation of unfolded or misfolded proteins and initiate three signaling pathways to restore ER homeostasis, also known as the unfolded protein response (UPR) [10, 11]. If ERS becomes too severe or persists for too long, the UPR will ultimately trigger apoptosis [10, 12]. ERS has been confirmed to play a key role in IVDD [13, 14]. The main components of the ECM include aggrecan and collagen II [15, 16]. The ECM participates in the metabolism and homeostasis of the IVD microenvironment by secreting various growth factors [17, 18]. ERS mediates ECM degradation, and the accumulation of ECM degradation products may, in turn, trigger ERS-related responses [19, 20]. This vicious cycle further exacerbates the deterioration of the intervertebral disc microenvironment, leading to pathological changes in the structure and function of the intervertebral disc [21, 22].

Protein kinase R-like endoplasmic reticulum kinase (PERK) is a key molecule in ERS. Downstream PERK activation leads to eIF2α/ATF4/CHOP expression, initiating cell apoptosis [23]. NOXA is a member of the BH3-only protein family and promotes mitochondrion-mediated apoptosis [24]. NOXA plays a key role in the pathogenesis of various cancers, including lung cancer [25], leukemia [26], prostate cancer [27], and multiple myeloma [28], making it an important factor in cancer development and a potential target for chemotherapy. However, the role and mechanism of NOXA in IVDD remain unknown.

In the present study, we aimed to determine the effects of NOXA in inducing cell death and ECM degradation upon sustained ERS in human NPCs. Our results showed that the PERK/NOXA/MCL-1 pathways are involved in the ERS-induced cell death and ECM degradation process in NPCs. Furthermore, through in vivo and in vitro experiments, our results showed the efficacy of silencing NOXA expression in treating IVDD, providing a therapeutic target for intervening IVDD from a pathophysiological standpoint.

## Results

### Identification and screening of key genes

**Fig. 1:**
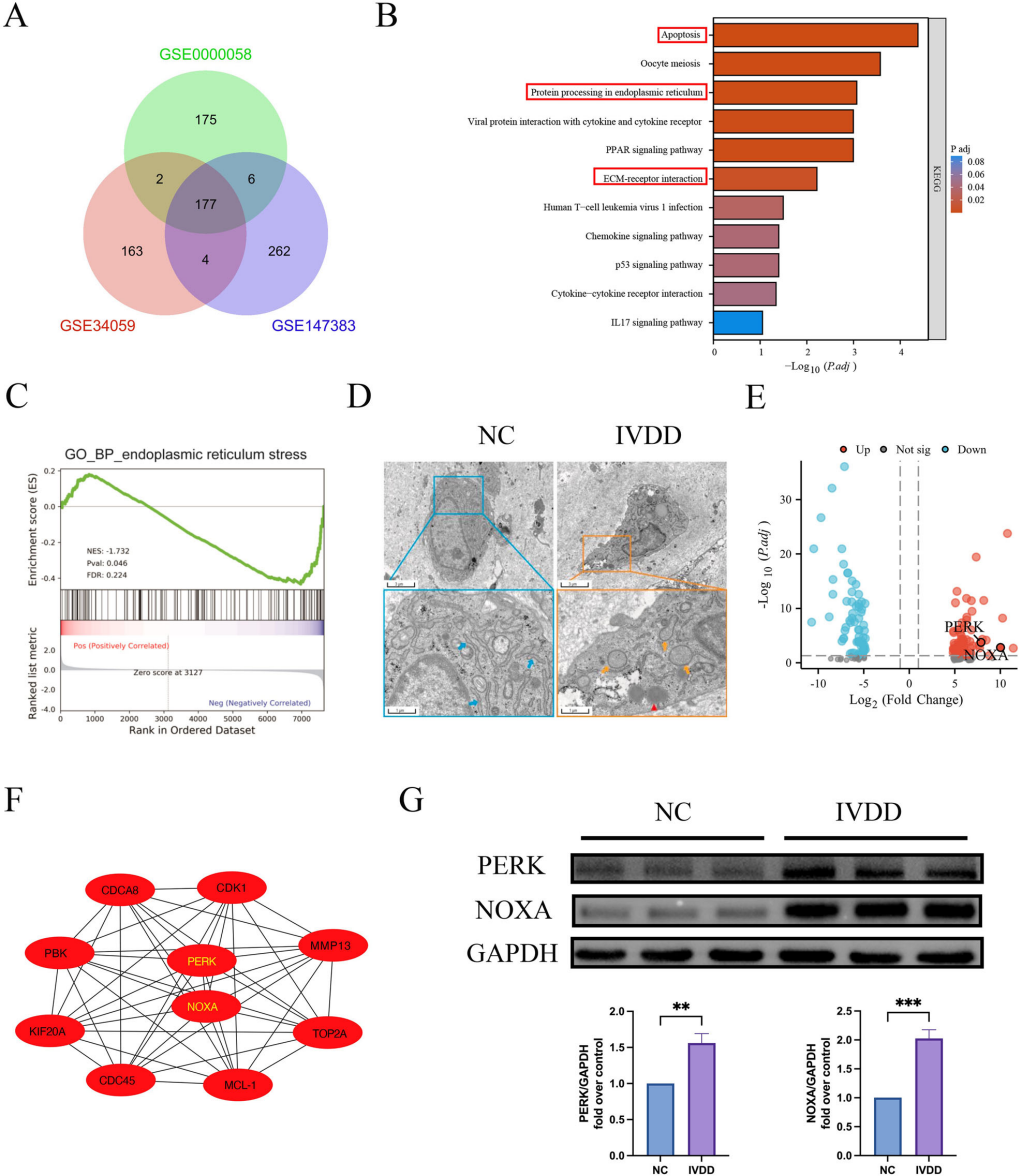
The expression of PERK and NOXA is elevated in human degenerated NPCs. **A** The Venn diagram of common DEGs among the three datasets. **B** the bar chart of KEGG pathway analysis, indicating that these DEGs are primarily enriched in the Apoptosis, Protein processing in the endoplasmic reticulum, and ECM-receptor interaction pathways. **C** The GSVA enrichment analysis revealed that the GO-BP of these differentially expressed genes is mainly enriched in endoplasmic reticulum stress. **D** Representative electron microscopy images of the nucleus pulposus tissue from the NC and IVDD groups. **E** the volcano plot of these DEGs, showing upregulated expression of PERK and NOXA. **F** the PPI network diagram of the top 10 key genes, placing PERK and NOXA at the core. **G** Western blot analysis and quantitative statistical analysis of PERK and NOXA proteins in the NC and IVDD groups. GAPDH was used as an internal control. Data are presented as mean ± SD, **P < 0.01, ***P < 0.001.

As shown in the Venn diagram (Fig. 1A), we screened 177 common differentially expressed genes (DEGs) from the GSE34059, GSE147383, and GSE0000058 datasets. We further explored the functions and pathways of these genes. Kyoto Encyclopedia of Genes and Genomes (KEGG) analysis revealed genes related to apoptosis, protein processing in the ER, and ECM-receptor interaction pathways (Fig. 1B). Gene set enrichment analysis revealed that these DEGs were primarily enriched in ERS (Fig. 1C). As shown in Fig. 1D, the ER appeared flat and cystic with a neat arrangement in the NC group. By contrast, in the IVDD group, the ER was significantly swollen and expanded, the normal lamellar folding structure disappeared, and apoptotic bodies were present.

Next, we identified two hub genes. Volcano plots showed that PERK and NOXA were significantly upregulated in the IVDD group (Fig. 1E). Protein–protein interaction network analysis revealed that PERK and NOXA are at the core (Fig. 1F). The results of Western blot analysis also revealed that PERK and NOXA were highly expressed in the IVDD group than in the NC group (Fig. 1G). These results indicate that ERS, PERK, and NOXA play an important role in IVDD.

### ERS promotes NPCs apoptosis and ECM degradation by increasing PERK and NOXA expression

Thapsigargin (TG), an ERS agonist, was used to further evaluate the role of ERS in IVDD. NPCs were treated with a series of TG concentrations, and the CCK-8 assay results showed that treatment with 500 nM TG for 12 h did not affect NPC viability (Supplementary Fig. S2A–D). These results indicate that TG can inhibit cell proliferation and promote apoptosis and can significantly increase PERK and NOXA expression.

**Fig. 2:**
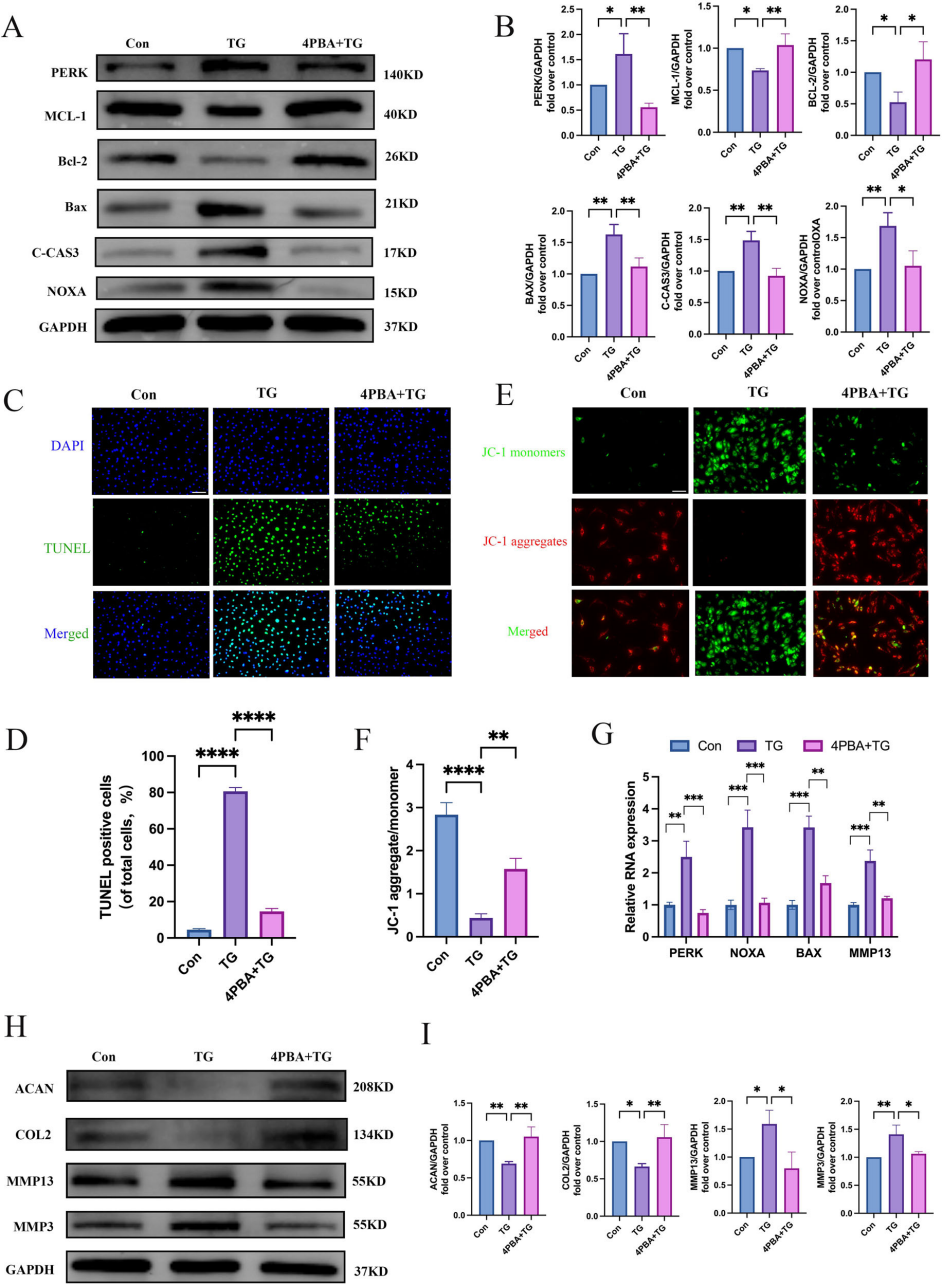
The ERS inhibitor 4-PBA alleviates TG-induced apoptosis and ECM degradation. **A**, **B** Western blot analysis of the expression levels of apoptosis-related proteins PERK, MCL-1, BCL-2, BAX, C-CAS3, and NOXA in NPCs after treatment with TG and TG + 4-PBA. GAPDH was used as an internal control. **C**, **D** Representative images of TUNEL staining and quantitative statistical analysis showing the proportion of TUNEL-positive cells in each group. The scale bar is 100 μm. **E**, **F** Representative images of JC-1 staining for mitochondrial membrane potential and quantitative statistical analysis. The scale bar is 100 μm. **G** mRNA levels of PERK, NOXA, BAX, and MMP13 in NPCs from each group. **H**, **I** Western blot analysis of the expression levels of ECM degradation-related proteins ACAN, COL2, MMP13, and MMP3 in NPCs after treatment with TG and TG + 4-PBA. GAPDH was used as an internal control. Data are presented as mean ± SD, *P < 0.05, **P < 0.01, ***P < 0.001, ****P < 0.0001.

Next, we treated the NPCs with the ERS inhibitor, 4-phenylbutyric acid (4-PBA). We observed that compared with TG, 4-PBA suppressed the expression of PERK, BAX, cleaved caspase 3 (C-CAS3), and NOXA while simultaneously increasing the expression of anti-apoptotic genes (MCL-1 and BCL-2) (Fig. 2A, B). The results of TUNEL staining showed that the percentage of positive staining cells was increased in the TG group than in the control group, and this increase could be partially reversed by 4-PBA (Fig. 2C, D). Moreover, as one of the characteristics of the early stages of apoptosis, mitochondrial membrane potential changes also participate in NPC apoptosis. The detection of mitochondrial membrane potential changes using JC-1 (Fig. 2E, F) showed that the JC-1 aggregates/monomers were decreased in the TG group than in the control group, and 4-PBA partially rescued this trend. Further highlighting the universal role of NOXA, experiments demonstrating Tunicamycin (TM)-induced ERS in NPCs would reinforce these findings (Supplementary Fig. S3 A, B).

In addition, we detected the expression of genes related to ECM degradation. Compared with the control group, the TG group had upregulated matrix metalloproteinase 3 (MMP3) and MMP13 expression and downregulated ACAN and COL2 expression. These trends could also be partially rescued by 4-PBA (Fig. 2G, H). Moreover, the polymerase chain reaction (PCR) results revealed that the TG group had significantly increased RNA expression levels of PERK, NOXA, BAX, and MMP13 compared with the control group, and 4-PBA could partially reverse this trend (Fig. 2I). Overall, these results indicate that the PERK pathway with enhanced NOXA levels in the mitochondria in response to TG may be responsible for the greater sensitivity of NPCs to ERS-inducing agents.

### GSK2606414 (GSK) can reverse NPC apoptosis and ECM degradation caused by NOXA overexpression

**Fig. 3:**
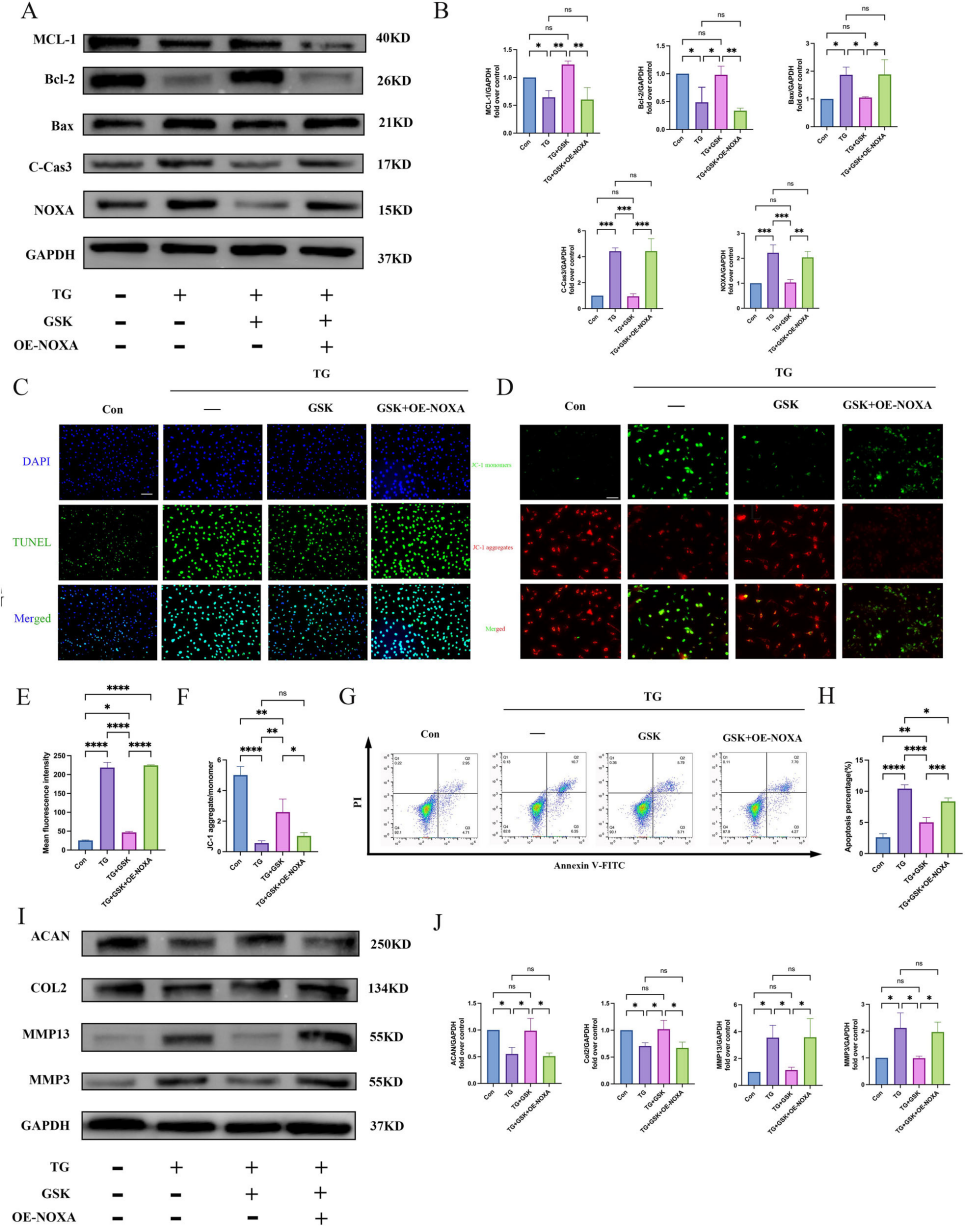
PERK inhibitor GSK rescues NPCs apoptosis and ECM degradation induced by NOXA overexpression. **A**, **B** Western blot analysis of the expression levels of apoptosis-related proteins MCL-1, BCL-2, BAX, C-CAS3, and NOXA in NPCs GAPDH was used as an internal control. **C**, **E** Representative images of TUNEL staining and quantitative statistical analysis showed each group’s proportion of TUNEL-positive cells. The scale bar is 100 μm. **D**, **F** Representative images of JC-1 staining for mitochondrial membrane potential and quantitative statistical analysis. The scale bar is 100 μm. **G**, **H** Representative flow cytometry scatter plot and quantitative analysis of apoptosis rates in NPCs of each group. **I**, **J** Western blot analysis of the expression levels of ECM degradation-related proteins ACAN, COL2, MMP13, and MMP3 in NPCs. GAPDH was used as an internal control. Data are presented as mean ± SD, *P < 0.05, **P < 0.01, ***P < 0.001, ****P < 0.0001.

The above results revealed that TG promoted PERK and NOXA transcription. Therefore, we hypothesized the involvement of TG in promoting NOXA transcription by activating PERK signal transduction, and we performed experiments to confirm our hypothesis. As a PERK inhibitor, GSK can significantly inhibit PERK expression [29–31]. The CCK-8 assay results indicated that treatment with 40 nM GSK for 6 h did not affect NPC viability (Supplementary Fig. S4A–D). We successfully constructed an overexpressed NOXA (OE-NOXA) in nucleus pulposus cells (NPCs), and qRT-PCR and Western blot analysis confirmed the overexpression of NOXA protein in these cells (Supplementary Fig. S5A–D). Similar to TG treatment, OE-NOXA can upregulate BAX and C-CAS3 expression while downregulating MCL-1 and BCL-2 expression. However, this trend can be partially reversed by GSK (Fig. 3A, B). Consistent with the above results, TUNEL staining (Fig. 3C, E) indicated that OE-NOXA promoted NPC apoptosis, whereas GSK inhibited NPC apoptosis. The mitochondrial membrane potential changes detected using JC-1 (Fig. 3D, F) showed a reduction in JC-1 aggregates/monomers in the TG and OE-NOXA groups compared with the control group, and this trend was partially rescued by GSK. Flow cytometry results (Fig. 3G, H) indicated that OE-NOXA promoted NPC apoptosis, whereas GSK inhibited NPC apoptosis.

Next, we evaluated ECM degradation. The results showed that compared with the control group, the TG and OE-NOXA groups had increased MMP3 and MMP13 expression and decreased ACAN and COL2 expression, which were also partially reversed by GSK (Fig. 3I, J). These data collectively suggest that the PERK pathway is responsible for regulating the extrinsic pathway of apoptosis upon ERS and plays a role in the pathway controlling NOXA levels.

### Knockdown of NOXA expression can rescue apoptosis and ECM degradation induced by TG

**Fig. 4:**
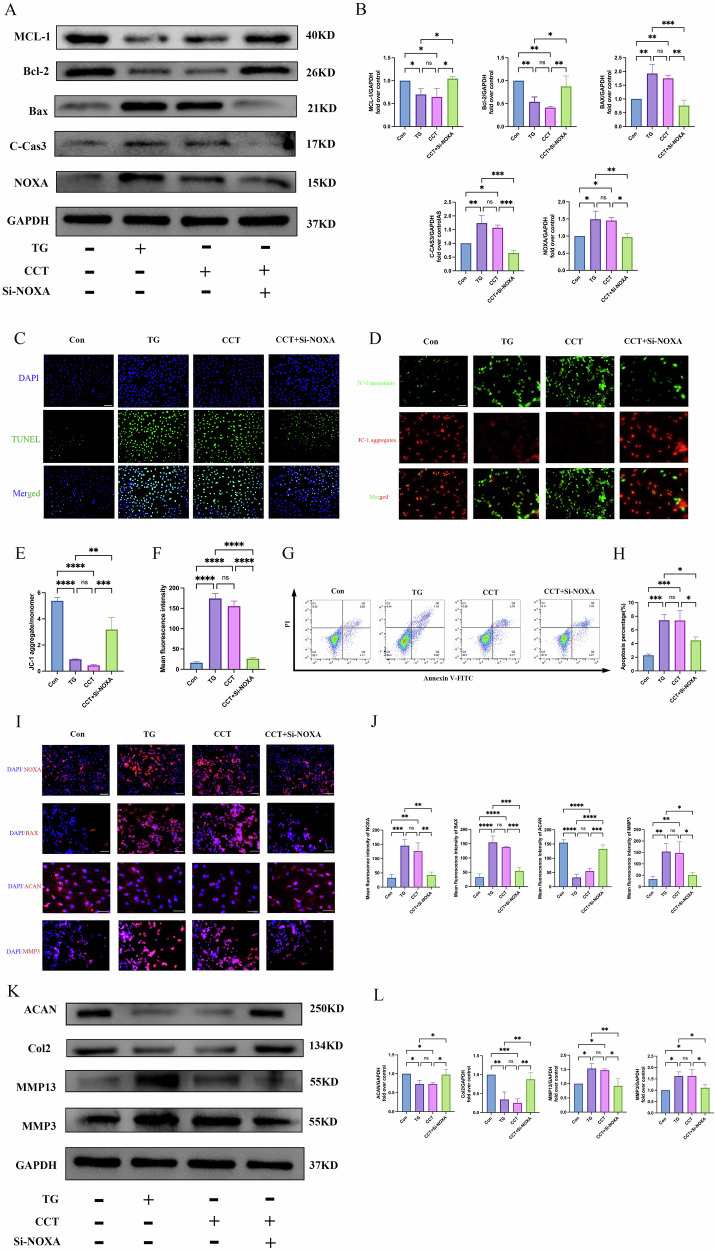
NOXA knockout rescues TG-induced apoptosis and ECM degradation. **A**, **B** Western blot analysis of the expression levels of apoptosis-related proteins MCL-1, BCL-2, BAX, C-CAS3, and NOXA in NPCs, GAPDH was used as an internal control. **C**, **F** Representative images of TUNEL staining and quantitative statistical analysis show each group’s proportion of TUNEL-positive cells. The scale bar is 100 μm. **D**, **E** Representative images of JC-1 staining for mitochondrial membrane potential and quantitative statistical analysis. The scale bar is 100 μm. **G**, **H** Representative flow cytometry scatter plot and quantitative analysis of apoptosis rates in NPCs of each group. **I**, **J** Representative images and quantitative statistical analysis of the expression of NOXA, BAX, ACAN, and MMP3 in each group by cellular immunofluorescence. The scale bar is 100 μm. **K**, **L** Western blot analysis of the expression levels of ECM degradation-related proteins ACAN, COL2, MMP13, and MMP3 in NPCs. GAPDH was used as an internal control. Data are presented as mean ± SD, *P < 0.05, **P < 0.01, ***P < 0.001, ****P < 0.0001.

To further observe the function of NOXA and its connection with ERS, we used small interfering RNA (Si-NOXA) to knock down NOXA expression. The qRT-PCR and Western blot analysis results confirmed that the NOXA protein was suppressed in NPCs (Supplementary Fig. S5E–H). CCT020312 (CCT) is a PERK agonist, and its CCK-8 analysis results showed that treatment with 1 μM CCT for 24 h did not affect the viability of NPCs (Supplementary Fig. S6A–D). The Western blot analysis results showed that the expression of BAX, C-CAS3, and NOXA was upregulated, whereas that of MCL-1 and BCL-2 was downregulated in the TG and CCT groups compared with the control group. These trends could be partially reversed by Si-NOXA (Fig. 4A, B). The TUNEL staining results showed that the percentage of positive staining cells was increased in the TG and CCT groups than in the control group, and this increase could be partially reversed by Si-NOXA (Fig. 4C, F). The detection of mitochondrial membrane potential changes using JC-1 showed that the TG and CCT groups had decreased JC-1 aggregates/monomers compared with the control group. Si-NOXA partially rescued this trend (Fig. 4D, E). The flow cytometry results revealed that the number of NPCs undergoing apoptosis was significantly higher in the TG and CCT groups than in the control group, and this increase could also be partially reversed by Si-NOXA (Fig. 4G, H). The cellular immunofluorescence results showed that the expression of NOXA, BAX, and MMP3 was significantly increased, whereas that of ACAN was significantly decreased in the TG and CCT groups compared with the control group. However, this trend was partially reversed by Si-NOXA (Fig. 4I, J).

Similarly, the expression of MMP13 and MMP3 was upregulated, whereas that of ACAN and COL2 was downregulated in the TG and CCT groups compared with those in the control group. These trends could also be partially reversed by Si-NOXA (Fig. 4K, L). The above results indicate that NOXA is an indispensable key molecule for PERK-induced apoptosis and ECM degradation.

### Suppressing NOXA expression ameliorates IVDD progression

**Fig. 5:**
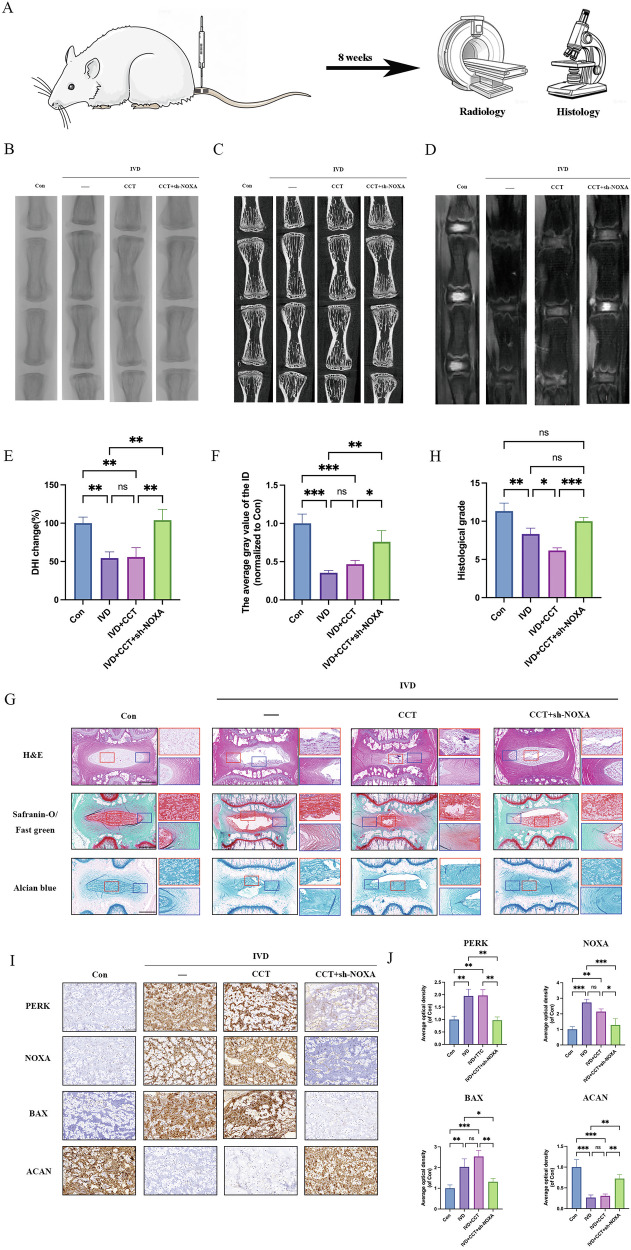
Imaging and histological analysis of rat coccygeal vertebrae tissue 8 weeks after NOXA gene knockout. **A** Schematic diagram of the overall process of animal experiments. **B**–**D** Representative X-ray, Micro-CT, and MRI T2WI images of rats in each group. **E** Percentage change in disc height index (DHI) of the tail vertebrae in each group of rats. **F** Histogram of the average gray values of the tail intervertebral discs in each group of rats. **G**, **H** Representative images and histological grade of H&E, Safranin O, and Alcian blue staining of the tail intervertebral disc tissue in each group. The scale bar is 500 μm. **I**, **J** Representative images and quantitative analysis histogram of immunohistochemical staining for PERK, NOXA, BAX, and ACAN in the NP tissue of rats from different treatment groups. The scale bar is 100 μm. Data are presented as mean ± SD, *P < 0.05, **P < 0.01, ***P < 0.001.

To evaluate the function of NOXA in IVDD progression, AAV9 was used to knock down NOXA expression in the NP tissue of the IVDD rat model. Imaging examination is a reliable method for assessing intervertebral disc degeneration and repair, and it is also indispensable in diagnosing spinal degenerative diseases. The overall process for the imaging and histopathological analysis of the rat tail vertebrae in each group is depicted in Fig. 5A. We obtained X-ray (Fig. 5B), micro-CT (Fig. 5C), and T2-weighted MRI (Fig. 5D) images of rat caudal vertebrae and calculated the percentage change in disc height index (DHI) for each group using X-ray images (Fig. 5E). The results showed that the DHI percentage of the IVDD and IVDD + CCT groups decreased to 55.5% and 54.8% of the control group, respectively (Fig. 5F). The sh-NOXA group restored the DHI% to some extent. T2-weighted MRI images revealed the water content within the NP tissue, with high signals indicating high water content in the NP, suggesting a mild degree of degeneration, whereas low signals indicated low water content in the NP, implying a more severe degree of degeneration. The MRI results showed that at 8 weeks, the intervertebral discs in the control group had intact structures with high signals (all classified as grade I). The intervertebral discs in the TG and TG + CCT groups had disordered structures with lower signals (classified as grade III–IV). The sh-NOXA group alleviated IVDD to some extent (classified as grade II–III) (Fig. 5F).

Hematoxylin and eosin (HE), Safranin O-fast green, and Alcian blue staining showed that in the control group at 8 weeks, the AF and NP boundaries were clear, and the structure was intact. In the IVDD and IVDD + CCT groups, the AF and NP boundaries were unclear, the NP was atrophied, and the AF layer structure was disrupted (Fig. 5G). Histological grading also reflected the same results (Fig. 5H). NOXA knockdown partially alleviated this degenerative trend. However, compared with the control group, there was a reduction in NP tissue and some disruption of the AF structure. Similarly, immunohistochemical staining of the NP tissue was used to detect PERK, NOXA, ACAN, and BAX expression (Fig. 5I, J). Compared with the control group, the IVDD and IVDD + CCT groups showed decreased ACAN expression and increased NOXA, BAX, and MMP13 expression. However, NOXA knockdown partially reversed this degenerative trend. Taken together, these results strongly suggest that knocking down NOXA expression in NPCs alleviates ERS-induced apoptosis and ECM degradation, and NOXA represents a potential therapeutic target for IVDD progression.

## Discussion

**Fig. 6:**
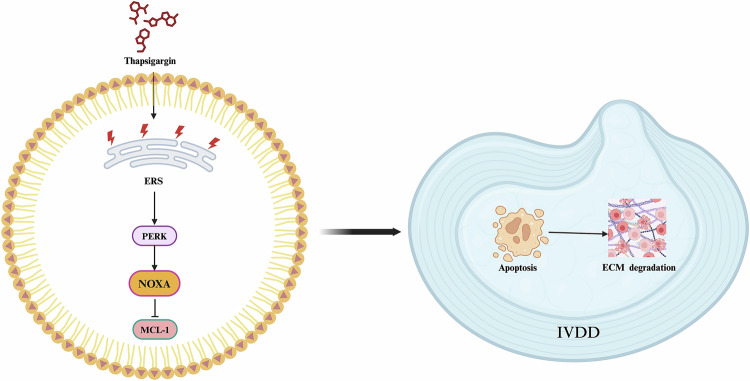
Schematic diagram of the overall mechanism in this study. TG induces ERS and leads to apoptosis of NPCs and ECM degradation through the PERK/NOXA/MCL-1 axis. Created in https://BioRender.com.

IVDD is the main cause of low back pain, affecting approximately 80% of individuals at some point in their lives and posing a significant economic burden on society [32, 33]. Conservative and surgical treatments of IVDD primarily aim to alleviate pain and restore stability, but these methods do not significantly slow the progression of IVDD [34, 35]. Current studies are investigating new strategies to restore the function of degenerative intervertebral discs and rebuild physiological balance by inhibiting inflammatory responses, preventing tissue aging, and increasing the ECM content [36]. In this study, we investigated the role of NOXA in cell death and the ECM degradation process activated by ERS in NPCs. Our results demonstrate that in NPCs, ERS-inducing agents activate cell apoptosis and ECM degradation processes through the PERK/NOXA/MCL-1 axis, exacerbating IVDD (Fig. 6). Thus, inhibiting NOXA expression can alleviate ERS-induced apoptosis and ECM degradation, thereby delaying IVDD progression. This study innovatively proposes the key role of NOXA in ERS-induced IVDD and identifies it as a potential therapeutic target.

IVDD is a degenerative disease characterized by cell apoptosis and ECM degradation, and ERS may be a potential pathogenic mechanism for IVDD [37]. The role of the ER in cells includes protein folding and lipid and ion transport, making it crucial for ECM synthesis [38]. In the NP tissue of patients with IVDD, the expression levels of ERS-related proteins and associated apoptosis markers (e.g., GRP78, CHOP, and caspase 12) are reportedly increased [6, 13]. The accumulation of reactive oxygen species (ROS) can induce ECM degradation and reduce the number of NPCs, thereby exacerbating IVDD progression [39]. This is related to the impact of ROS on disulfide bond formation and proper protein folding, further triggering ERS [40]. Moreover, the ER–mitochondrial Ca^2+^ crosstalk may play an important role in ROS-induced ERS [41]. Tauroursodeoxycholic acid, an FDA-approved bile acid with chaperone properties, has been found to reduce NPC apoptosis induced by ERS [42]. The present study also found that TG can cause an increase in intracellular ROS, which can be alleviated by 4-PBA, another FDA-approved chemical chaperone that can alleviate IVDD by inhibiting ERS [20, 43].

Under continuous ERS, cells initiate a proapoptotic UPR pathway to eliminate existing cells, thus exacerbating the condition, a phenomenon known as proapoptotic UPR. Therefore, ERS is an important pathway that leads to cell apoptosis [44]. The present study innovatively discovered that NOXA can bridge ERS and apoptosis, providing a new therapeutic target for IVDD. The role of NOXA in inducing apoptosis has been extensively studied in various cancers [45–47]. However, few studies have investigated its role in IVDD. Studies have shown that NOXA initiates apoptosis through its ubiquitination, utilizing its BH3 domain to translocate MCL-1 to the mitochondria, where it is then phosphorylated and ubiquitinated, promoting its degradation by the proteasome and thereby reducing MCL-1 levels to induce apoptosis [48]. To investigate the relationship between NOXA and MCL-1 in IVDD, we conducted in vitro and in vivo experiments. We found that NOXA overexpression led to a decrease in MCL-1 expression levels, whereas NOXA knockdown increased MCL-1 expression levels. This finding suggests that in IVDD, NOXA can also inhibit MCL-1 expression, thereby promoting apoptosis. Antitumor drugs targeting NOXA are widely applied in various cancers, including BH3-mimetics [49–51] and NOXA-like mimetics [52, 53], which provide an excellent precedent for the future development of NOXA-targeted therapies for IVDD.

The physiological function of the NP relies on its ECM that is rich in type II collagen, elastin, and proteoglycans, which can disperse and transfer the pressure exerted on the spine [54]. The AF comprises alternating type I collagen fibers, and its function is to prevent the NP from protruding under compression during spinal movement. As a type of hyaline cartilage, the CEP has an ECM primarily composed of proteoglycans and collagen fibers, with uniform thickness [55, 56]. The avascular and denervated intervertebral disc relies on the CEP for the delivery of nutrients and the removal of metabolic waste to maintain its structural and functional stability. Therefore, ensuring the health of NPCs is crucial for the overall health of the intervertebral disc. As IVDD progresses, the activity of MMPs and a disintegrin and metalloproteinase with thrombospondin motifs (ADAMTS) increases, and these proteases degrade type II collagen and proteoglycans, thereby exacerbating IVDD [57, 58]. The present study also focused on ECM degradation, where ERS leads to increased MMP 3 and MMP 13 expression, resulting in a decrease of ACAN and COL2. This finding indicates that ERS-mediated IVDD also functions through ECM degradation by metalloenzymes.

The activation of the PERK pathway typically initiates downstream signaling through ATF4 and its pro-apoptotic target CHOP. Studies have demonstrated that mesenchymal stem cell-derived exosomes suppress ER stress (PERK/eIF2α/ATF4/CHOP pathway), thereby reducing nucleus pulposus (NP) cell death and ameliorating intervertebral disc degeneration (IDD) [6]. Teng et al. reported that fucoxanthin upregulates Sirt1, inhibits the PERK-eIF2α-ATF4-CHOP signaling pathway, alleviates ER stress, suppresses apoptosis, and attenuates IDD in rat models [59].

Recent evidence suggests that the three branches of the unfolded protein response (UPR) — PERK, IRE1, and ATF6—may form a dynamic signaling network through crosstalk. For instance, during chronic ER stress, the IRE1/XBP1s pathway has been shown to sustain PERK expression [60]. Furthermore, under ER stress conditions, PERK-mediated transcriptional downregulation of the miR-424(322)-503 cluster inhibits the optimal activation of IRE1 and ATF6 pathways [61]. Notably, earlier studies revealed that XBP1 splicing accumulation is dependent on PERK activation [62], while PERK can also suppress the expression of a specific subset of XBP1-ATF6 targets [63]. Collectively, these findings underscore the intricate crosstalk among the three UPR branches.

Inevitably, this study has some limitations. RNA interference (RNAi) technology, including shRNA, inherently carries the risk of off-target effects due to partial sequence homology with unintended transcripts. Although this study confirmed the importance of NOXA in the PERK/NOXA/MCL-1 axis, it did not fully explore the specific mechanism of action and the upstream and downstream regulatory relationships of this pathway. Moreover, although this study provided new targets for IVDD treatment, these therapeutic strategies are still in the preliminary exploration stage and require further preclinical and clinical studies to validate their efficacy and safety.

## Conclusion

This study confirmed the role of ERS in promoting IVDD, which has been revealed by previous studies. Mechanistically, ERS exacerbates NPC apoptosis and ECM degradation via the PERK/NOXA/MCL-1 pathways. Targeting NOXA expression represents a promising therapy for IVDD. These findings provide a foundation for future research on the molecular mechanisms and therapeutic strategies of IVDD.

## Materials and methods

### Bioinformatics analysis

Our research group previously performed single-cell sequencing (GSE0000058) and intersected the differentially expressed genes (DEGs) with those found in IVD-related transcriptomic sequencing results from the GEO database (GSE34059 and GSE147383). We used the ggplot2 (version 3.3.6) and Venn Diagram (version 1.7.3) to plot a Venn diagram of these DEGs. Subsequently, we utilized the ggplot2 (version 3.3.6) to create a volcano plot for these DEGs. The protein–protein interaction (PPI) network was based on the STRING website (https://string-db.org/) and visualized in Cytoscape (version 3.10.2). Finally, we performed KEGG enrichment analysis on these DEGs using the ggplot2 (version 3.3.6) and visualized the results.

### Nucleus pulposus cells isolation and culture

The Ethics Committee of Qingdao University Affiliated Hospital approved all experimental protocols. Under the patients’ informed consent, normal NPCs were obtained from patients with idiopathic scoliosis, and degenerated NPCs were obtained from patients with lumbar disc herniation (LDH). The NP tissue removed during surgery was cut into tissue fragments, digested for 2 h at 37 °C with shaking using collagenase II (Gibco, New York, USA), and then filtered using a mesh filter. The filtrate was cultured in DMEM/F12 medium (Gibco, New York, USA) supplemented with 10% fetal bovine serum (Procell, Wuhan, China) and 1% penicillin-streptomycin (S110JV, BasalMedia Co., Ltd, shanghai, China). The medium was changed for the first time on the 5th day of culture and every 3 days after that. Cells from the 2nd and 3rd generations were used for subsequent experiments.

### Animal experiment

Twenty male Sprague-Dawley (SD) rats (8-week-old) were purchased from Beijing Vital River Laboratory Animal Co., Ltd. and housed under a 12-hour light/dark cycle with the temperature maintained at 20–25 °C and humidity at 40–60% for a 1-week acclimation period. The rats were randomly allocated to either the control group or experimental groups using simple randomization, with investigators and data analysts blinded to group assignments throughout the study. The Ethics Committee of Qingdao University Affiliated Hospital approved the use of animals. Rats were anesthetized with 2% (w/v) pentobarbital (40 mg/kg, Merk, Darmstadt, Germany). The SD rats were randomly assigned into groups using randomization methods. Throughout the entire experimental process, investigators involved in data collection and outcome assessment remained blinded to group allocation. The control group rats received no special treatment. In the model group (*n* = 5), a 22 G needle punctured the rat’s caudal intervertebral disc (Co7/8, Co8/9, and Co9/10) with X-ray guidance to a depth of 4 mm. The needle was rotated once and remained in place for 1 min after entering the intervertebral disc [64]. For the CCT group (*n* = 5), CCT020312 (2 mg/kg) was administered via intraperitoneal injection once daily for 4 weeks. In the shNOXA group (*n* = 5), after modeling, 5 μL of AAV9-shNOXA with a titer of 1 × 10¹² vg/mL (GeneChem, Shanghai, China) was injected into the center of the intervertebral disc nucleus using a microsyringe (Gaoge, Shanghai, China).Some of NP tissues were fixed with paraformaldehyde (4%), whereas the remaining were frozen immediately in liquid nitrogen.

### Western blot analysis

Total protein was extracted from NPCs using a total protein extraction kit (BC3710, Solarbio, Beijing, China), and the protein concentration was detected using a BCA kit (Epizyme, Shanghai, China). Proteins were separated using a 6–15% gel and transferred onto PVDF membranes. The membranes were then blocked with 5% skim milk diluted in TBST for 1 h at room temperature, followed by three washes with TBST, each for 10 min. The first antibodies were incubated overnight at 4 °C with the following dilutions: NOXA (ab222852, 1:1000, Abcam, Cambridge, UK), PERK (bs-2469R, 1:1000, Bioss, Beijing, China), BAX (bs-0127R, 1:1000, Bioss, Beijing, China), BCL-2 (bs-0032R, 1:2000, Bioss, Beijing, China), MCL-1 (16225-1-AP, 1:1000, Proteintech, Wuhan, China), MMP3 (17873-1-AP, 1:1000, Proteintech, Wuhan, China), MMP13 (18165-1-AP, 1:1000, Proteintech, Wuhan, China), Cleaved Caspase-3 (25128-1-AP, 1:1000, Proteintech, Wuhan, China), ACAN (bs-1223R, 1:1000, Bioss, Beijing, China), COL2 (bs-5881R, 1:1000, Bioss, Beijing, China), GAPDH (10494-1-AP, 1:5000, Proteintech, Wuhan, China). After washing with TBST, the membranes were incubated with a secondary antibody conjugated to HRP (Proteintech, Wuhan, China) for 1 h at room temperature. Following washing with TBST, the protein bands were detected using a chemiluminescent reagent (SQ203, Epizyme, Shanghai, China) in the imaging system (UVITEC, Cambridge, UK). GAPDH was used for normalization. Uncropped immunoblot gels are shown in supplementary file.

### Quantitative real-time polymerase chain reaction (qRT-PCR)

Total RNA was extracted from NPCs using an RNA extraction kit (Vazyme, Nanjing, China), and the concentration of RNA was determined using Nanodrop One (Thermo Fisher Scientific, Waltham, USA). The total RNA was then reverse transcribed into cDNA using a reverse transcription kit (Vazyme, Nanjing, China) on an instrument (Bio-Rad, Hercules, USA). The primers used for qPCR were synthesized by Sangon Biotech (shanghai, China) and are listed in Table 1. GAPDH acted as an endogenous control, and the expression of target gene mRNA was calculated using the 2^−ΔΔCT^ method.

**Table 1:** Primers used in qRT-PCR.

Gene	Forward primers	Reverse primers
GAPDH	AATCCCATCACCATCTTCC	GAGTCCTTCCACGATACCAA
NOXA	CTCAGGAGATTTGGAGACAA	CTGCCGGAAGTTCAGTTT
PERK	TTGTCGCCAATGGGATAG	CAGTCAGCAACCGAAACC
BAX	TTTTGCTTCAGGGTTTCATC	GGGACATCAGTCGCTTCAGT
CAS3	GAATATCCCTGGACAACA	TTAGAAACATCACGCATC

### Transmission electron microscopy (TEM)

The nucleus pulposus tissue extracted during surgery was fixed overnight in 2.5% glutaraldehyde (Biosharp, Hefei, China), then fixed in 1% osmium tetroxide (Electron Microscopy Sciences, Hatfield, USA), dehydrated in a gradient of ethanol, and embedded in Epon-812 resin. The sample was cut into 60–80 nm thin sections using an ultramicrotome. The sections were stained with uranyl acetate (Electron Microscopy Sciences, Hatfield, USA) and lead citrate (Electron Microscopy Sciences, Hatfield, USA), and images were captured using a HT7700 transmission electron microscope (HITACHI, Tokyo, Japan). The micrographs were analyzed using Volocity 3D image analysis software (PerkinElmer, Waltham, USA).

### Terminal deoxynucleotidyl transferase dUTP nick end labeling (TUNEL) staining

The cells were fixed with 4% paraformaldehyde (Beyotime, Shanghai, China) at room temperature for 1 h, followed by treatment with 0.5% Triton X-100 (Beyotime, Shanghai, China) for 30 min. The cells were washed with PBS (Beyotime, Shanghai, China) 3 times, each for 5 min. TUNEL reaction mixture (containing TdT enzyme and dUTP labeled with fluorescence) (Roche, Basel, Switzerland) was added and incubated at room temperature for 1 h. The cell nuclei were stained with DAPI (Beyotime, Shanghai, China) for 5 min. The samples were observed under a fluorescence microscope (Keyence, Osaka, Japan), and images were captured.

### JC-1 staining

The cells were seeded into a 24-well plate at a density of 5 × 10^5^ cells/mL and cultured overnight in the cell incubator. The cells in each group were gently washed with PBS for 3*5 min. Then, 200 μL of 5 μg/mL JC-1 staining solution (Med Chem Express, Shanghai, China) was added to each well, and the cells were incubated for 15 min. ROS detection follows the same steps as the JC-1 staining procedure. After washing with PBS, the cells were observed under a fluorescence microscope (Keyence, Osaka, Japan) with at least 3 random fields of view from each well.

### Plasmid construction, siRNA constructs and transfection

To overexpress NOXA, plasmids carrying NOXA gene fragments were transfected into NPCs using lentivirus. OE-NOXA was also synthesized by GeneChem (Shanghai, China) and transfected according to the experimental protocol. To knockdown NOXA expression using short interfering RNA (siRNA), Si-NOXA was synthesized by Genechem (Shanghai, China) and transfected strictly according to the experimental protocol using Lipofectamine 2000 (Invitrogen, Carlsbad, USA). After transfection with Si-NOXA and OE-NOXA, the cells were observed under a fluorescence microscope (Keyence, Osaka, Japan) to confirm a high transfection efficiency, and the NPCs were then used for subsequent experiments.

### Immunofluorescence analysis

Cells were inoculated into a culture plate (TCP040006, Jet Biofil, China) and cultured overnight in an incubator. Cells were fixed with 4% paraformaldehyde (Beyotime, Shanghai, China) at room temperature for 30 min, followed by permeabilization with 0.2% Triton X-100 (Beyotime, Shanghai, China) at room temperature for 15 min. The cells were washed with PBS for 3 times, 5 min each. Incubation with 5% fetal bovine serum (Gibco, New York, USA) was carried out at 37 °C for 1 h, after which first antibodies were added: NOXA (ab222852, 1:100, Abcam, Cambridge, UK), ACAN (bs-1223R, 1:100, Bioss, Beijing, China), BAX (bs-0127R, 1:100, Bioss, Beijing, China), MMP3(17873-1-AP, 1:100, Proteintech, Wuhan, China) and incubated overnight at 4 °C. After washing with PBS, fluorescently labeled secondary antibodies (KTD109, Abbkine, Wuhan, China) were added and incubated at 37 °C for 1 h. The cell nuclei were stained with DAPI (Beyotime, Nantong, China) for 5 min. The samples were observed under a fluorescence microscope (Keyence, Osaka, Japan), and images were captured.

### Cell viability assay

Cells were seeded into each well of a 96-well plate at a density of 1*10^5^ cells and cultured for 24 h. After treatment with various concentrations of TG for 24 h, 10% CCK-8 reagent (Beyotime, Shanghai, China) was added to each well, and the plate was incubated for 1 h at 37 °C in the dark. The absorbance at 450 nm was measured using an enzyme-labeled instrument (Molecular Devices, Sunnyvale, USA), and the cell viability was calculated.

### Imaging analysis

Rat IVDD conditions were assessed using X-ray, micro-CT, and micro-MRI. At 8 weeks post-operation, the rats were anesthetized and immobilized, and sagittal T2-weighted images (T2WI) of the rat tail vertebrae were captured using a 7.0 T magnetic resonance scanner (Bruker, Billerica, USA). The gray scale values of the intervertebral disc in the MRI T2WI images were measured using ImageJ (version: 1.53t, author: Wayne Rasband) to reflect the water content of the nucleus pulposus (NP) tissue. Following this, the rats were euthanized, and the isolated tail vertebrae were fixed with 4% paraformaldehyde. The tail vertebrae were scanned using micro-CT and X-ray (Bruker, Billerica, USA) to obtain images. The change rate of the Disc Height Index (DHI) [65] was measured and calculated using ImageJ (version: 1.53t, author: Wayne Rasband), with the specific measurement method as shown in Supplementary Fig. S1.

### Histological staining

After euthanizing the rats, their coccygeal vertebrae were collected and placed in 4% paraformaldehyde (Beyotime, Shanghai, China) for fixation for 48 h, followed by decalcification in EDTA decalcifying solution (Beyotime, Shanghai, China) for one month. The specimens were then dehydrated with a gradient of alcohol and cleared with xylene before being embedded in paraffin and sectioned. The sections were processed according to the standard procedures for HE, Safranin O/Fast green, and alcian blue (Solarbio, Beijing, China).

### Immunohistochemistry

The sections were placed in 0.01 mol/L citrate antigen retrieval buffer (Beyotime, Shanghai, China) and microwaved for 15 min. Then, the sections were incubated in 3% hydrogen peroxide (Merk, Darmstadt, Germany) for 10 min to block endogenous peroxidase activity, followed by incubation with 5% fetal bovine serum (Gibco, New York, USA) for 30 min to block non-specific binding sites. First antibodies were added, including NOXA (ab222852, 1:100, Abcam, Cambridge, UK), PERK (bs-2469R, 1:50, Bioss, Beijing, China), MMP13 (18165-1-AP, 1:50, Proteintech, Wuhan, China), ACAN (bs-1223R, 1:50, Bioss, Beijing, China), and incubated overnight in a 4 °C refrigerator. Subsequently, the sections were incubated with horseradish peroxidase-conjugated secondary antibodies (Proteintech, Wuhan, China) at room temperature for 1 h, followed by the addition of DAB chromogenic solution (Proteintech, Wuhan, China), and then incubated with hematoxylin staining solution (Beyotime, Shanghai, China) at room temperature for 5 min. The sections were then observed under a microscope (Keyence, Osaka, Japan) and documented with photographs.

### Flow cytometry

The cells were digested and collected using 0.25% trypsin without EDTA (Gibco, New York, USA), washed with PBS, and resuspended in Binding Buffer. Annexin V–fluorescein isothiocyanate/Propidium Iodide (Elabscience, Wuhan, China) was used to detect cell apoptosis. Finally, the samples were detected using a flow cytometer Attune NXT (Thermo Fisher Scientific, Waltham, USA) and analyzed using FlowJo (version 10.8.1, Becton Dickinson & Company, New York, USA).

### Statistical analysis

Experiments are conducted at least three times, and the data are expressed as the mean ± standard deviation (SD). For three or more groups, one-way ANOVA is used for continuous variables, followed by the Tukey post-hoc test for multiple comparisons. An independent sample t-test is used for continuous variables for two groups, and *P* < 0.05 is considered statistically significant. Statistical analysis and graphing were performed using GraphPad Prism (version 10.2.3).

## Supplementary information

Supplementary results

Uncropped gels image for Western Blot

## Data Availability

The data presented in the study are deposited in the China National Gene Bank repository, accession number CNP0002664 (https://db.cngb.org/search/project/CNP0002664/) and Gene Expression Omnibus (GEO) datasets (https://www.ncbi.nlm.nih.gov/geo/).
