# Supplementary material for: NOXA exacerbates endoplasmic-reticulum-stress-induced intervertebral disc degeneration by activating apoptosis and ECM degradation
Source: Cell Death Discov. 2025 May 28;11:257. doi: 10.1038/s41420-025-02539-0 (PMC12119965; doi:10.1038/s41420-025-02539-0)
Supplement: Supplementary file 1 — Supplementary results [file 41420_2025_2539_MOESM1_ESM.docx]

**NOXA exacerbates endoplasmic-reticulum-stress-induced intervertebral disc degeneration by activating apoptosis and ECM degradation**

Zhiming Liu^1^, Hui Lu^1^, Xianjuan Zhang^2^, Shuai Tang^1^, Antao Lin^1^, Shuo Han^1*^, Xuexiao Ma^1*^

**Supplementary results**

**
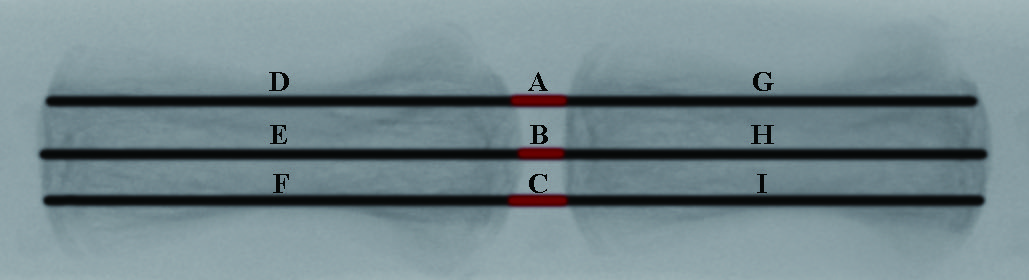
**

**Figure S1 The specific measurement method for the Disc Height Index (DHI).** DHI=2×(A+B+C) / (D+E+F+G+H+I)


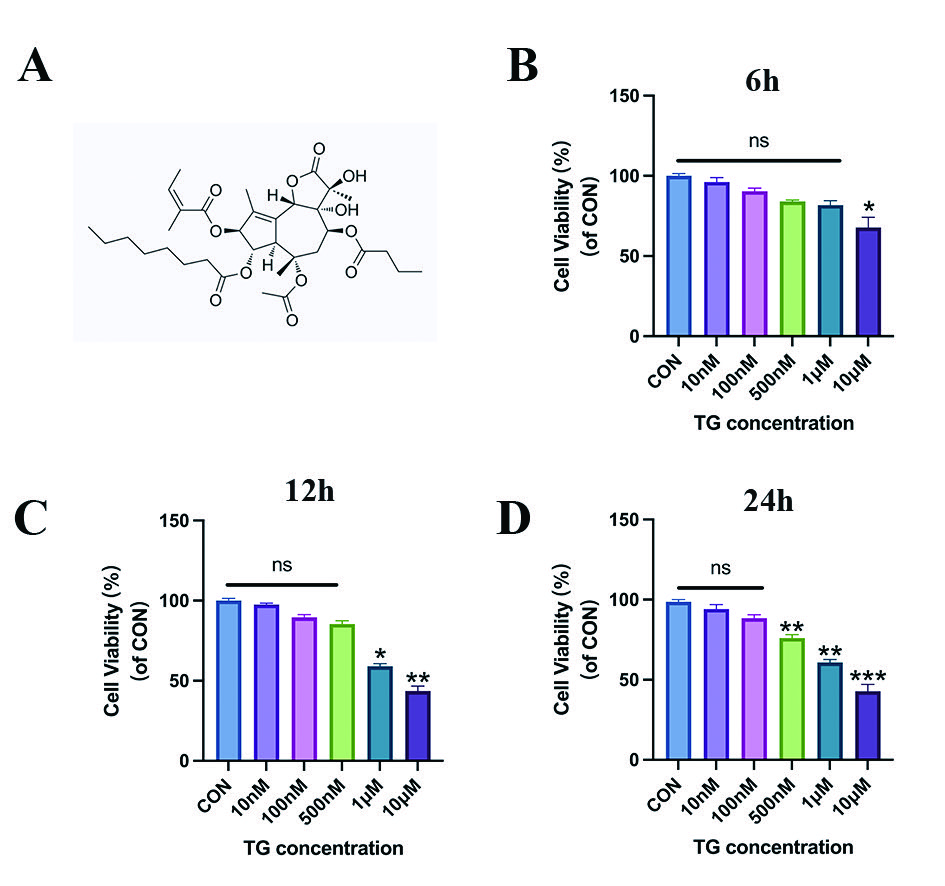


**Figure S2 Chemical structure of TG and its effect on the viability and proliferation of NPCs.** (**A**) Chemical structure of TG. (**B, C, and D**) The effects of different concentrations of TG on the proliferation of NPCs at 6 hours, 12 hours, and 24 hours were measured using the CCK-8 assay. Data are presented as mean ± SD, *P < 0.05, **P < 0.01, *** P< 0.001.

**
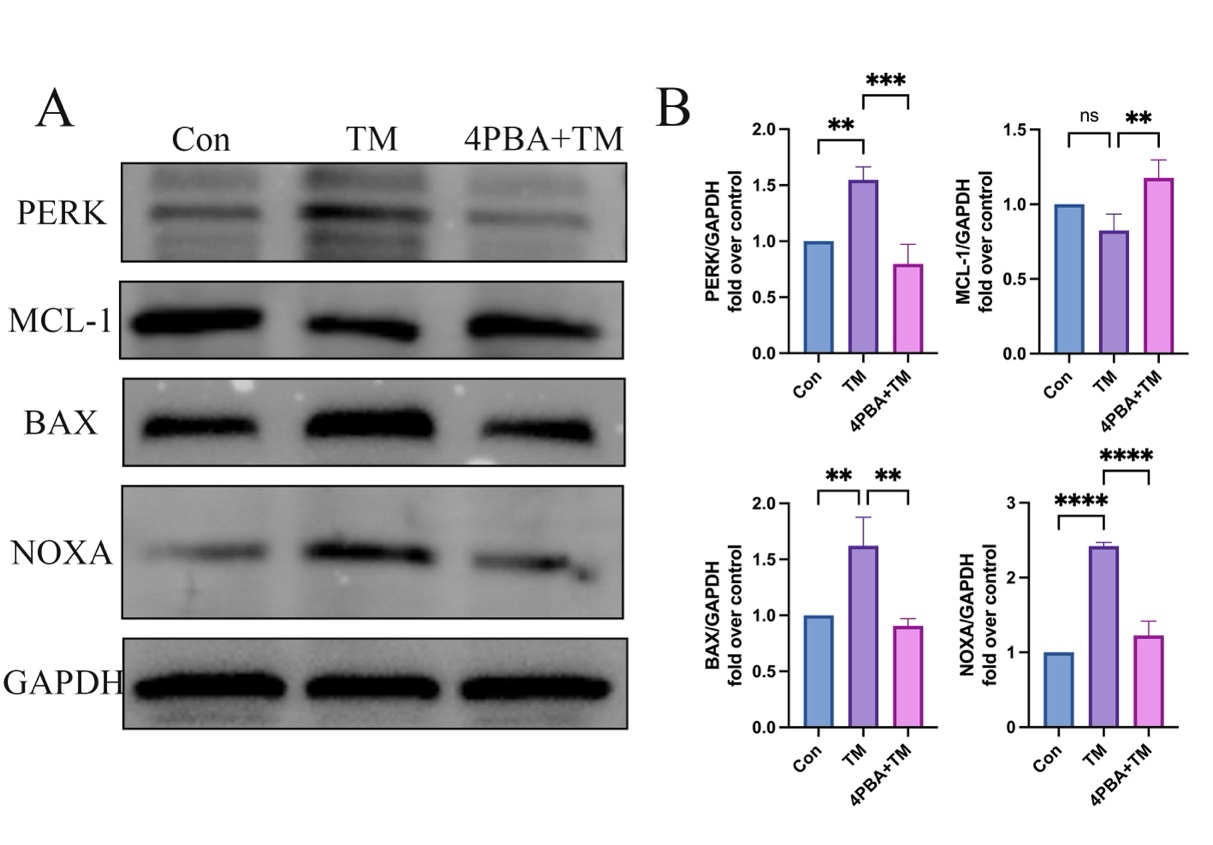
**

**Figure S3 Tunicamycin (TM)-induced ERS and apoptosis in NPCs were partially rescued by the ERS inhibitor 4-PBA. (A, B)** Western blot analysis of the expression levels of apoptosis-related proteins PERK, MCL-1, BAX, and NOXA in NPCs, GAPDH was used as an internal control. Data are presented as mean ± SD, **P < 0.01, *** P< 0.001, **** P < 0.0001.

**
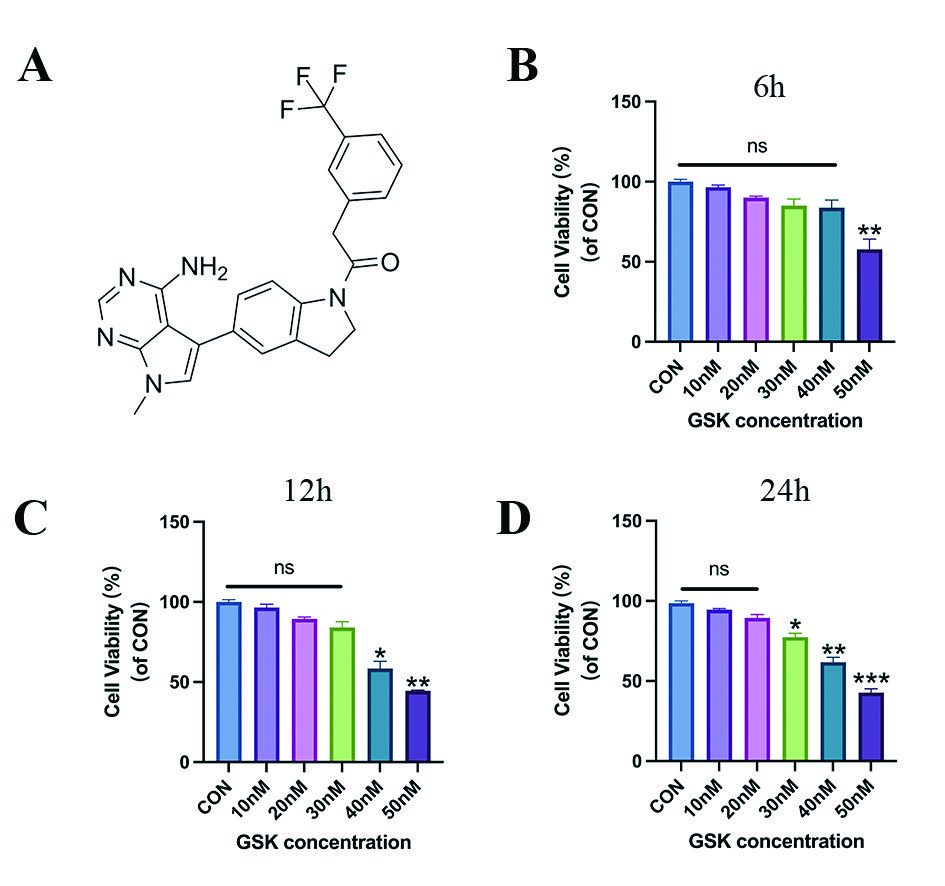
**

**Figure S4 Chemical structure of GSK and its effect on the viability and proliferation of NPCs.** (**A**) Chemical structure of GSK. (**B, C, and D**) The effects of different concentrations of GSK on the proliferation of NPCs at 6 hours, 12 hours, and 24 hours were measured using the CCK-8 assay. Data are presented as mean ± SD, *P < 0.05, **P < 0.01, *** P< 0.001.


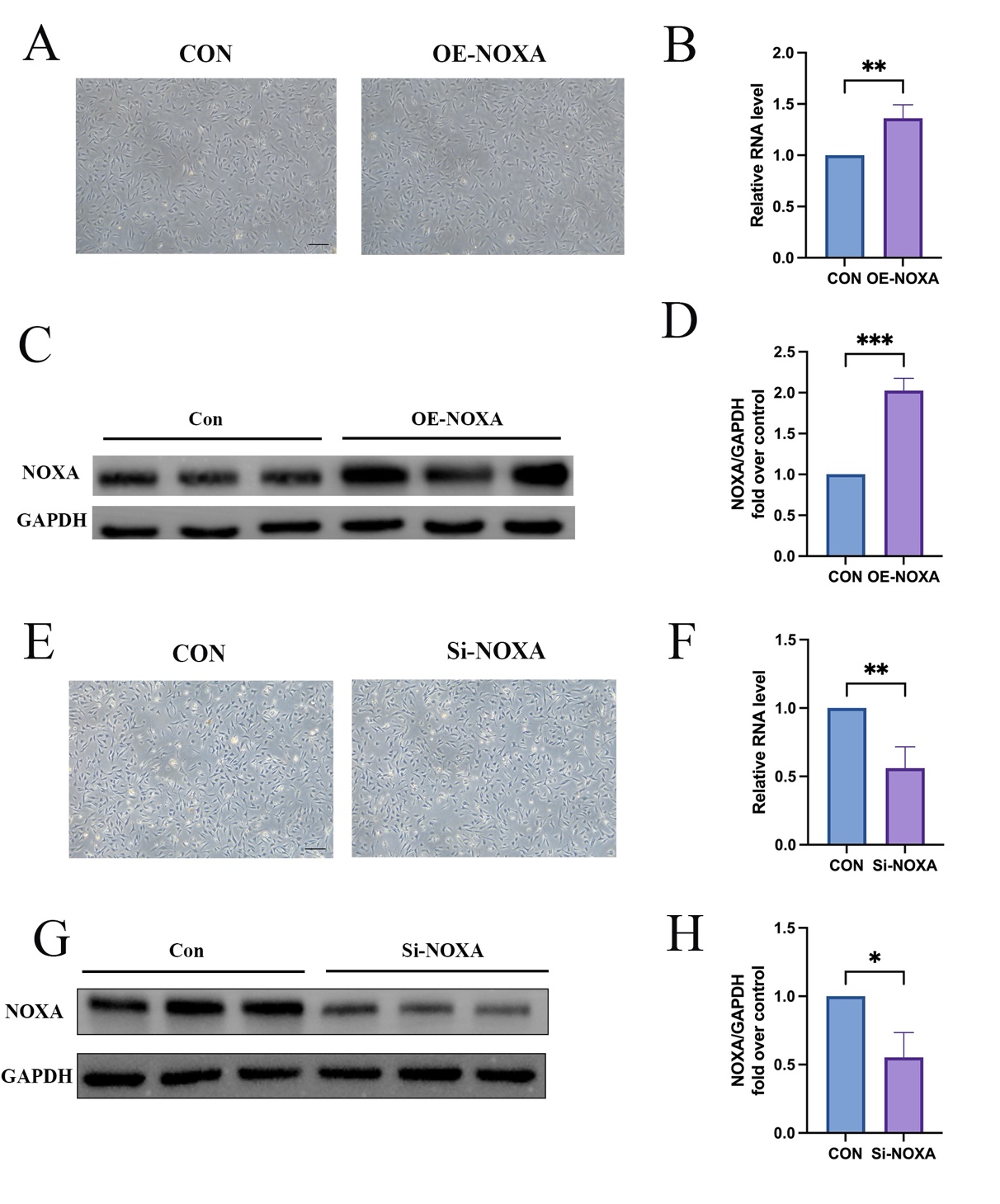


**Figure S5 Validate the transfection efficiency of NOXA overexpression and knockdown at the RNA and protein levels.** (**A and E**) The white light image of the cells after transfection showed no significant abnormalities in cell morphology and viability. The scale bar is 100μm. (**B and F**) qRT-PCR detection of NOXA mRNA expression in NPCs after OE-NOXA and Si-NOXA transfection. (**C and D**) Western blot analysis of NOXA in OE-NOXA transfected NPCs. (**G and H**) Western blot analysis of NOXA in Si-NOXA transfected NPCs. GAPDH was used as an internal control. Data are presented as mean ± SD, **P < 0.01.

**
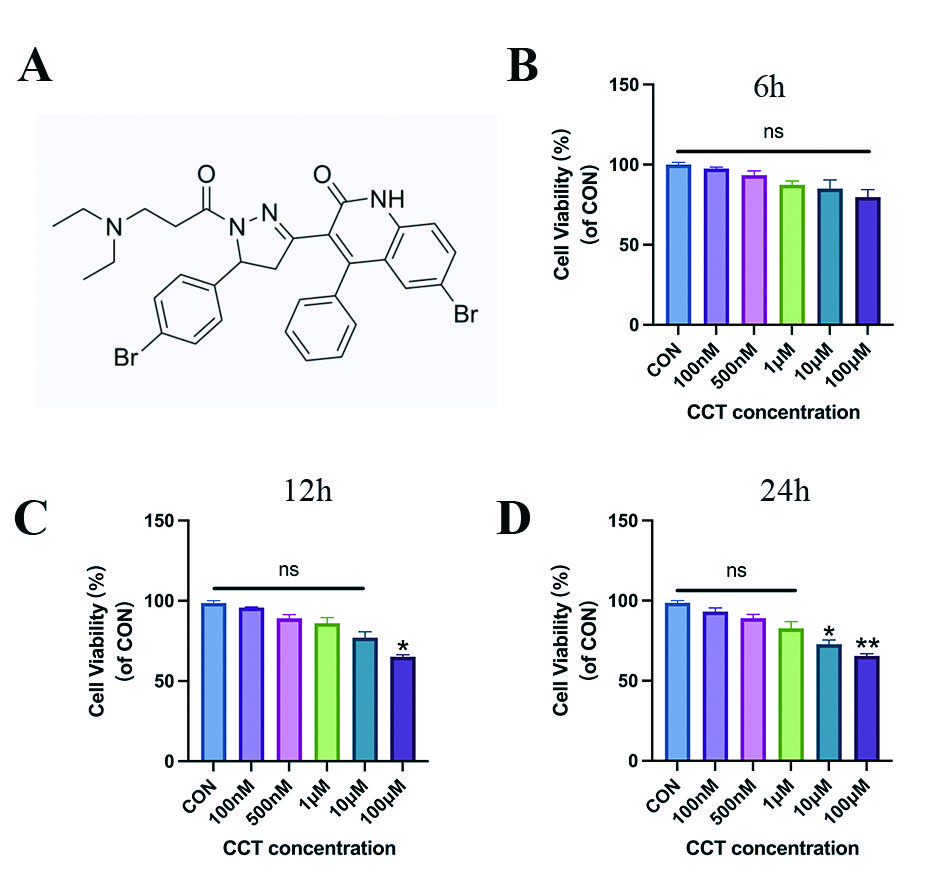
**

**Figure S6 Chemical structure of CCT and its effect on the viability and proliferation of NPCs.** (**A**) Chemical structure of CCT. (**B, C, and D**) The effects of different concentrations of CCT on the proliferation of NPCs at 6 hours, 12 hours, and 24 hours were measured using the CCK-8 assay. Data are presented as mean ± SD, *P < 0.05, **P < 0.01, *** P< 0.001.
