# Supplementary material for: NOXA exacerbates endoplasmic-reticulum-stress-induced intervertebral disc degeneration by activating apoptosis and ECM degradation
Source: Cell Death Discov. 2025 May 28;11:257. doi: 10.1038/s41420-025-02539-0 (PMC12119965; doi:10.1038/s41420-025-02539-0)

Uncropped gels for Western Blots in Figure 1

Figure 1-G

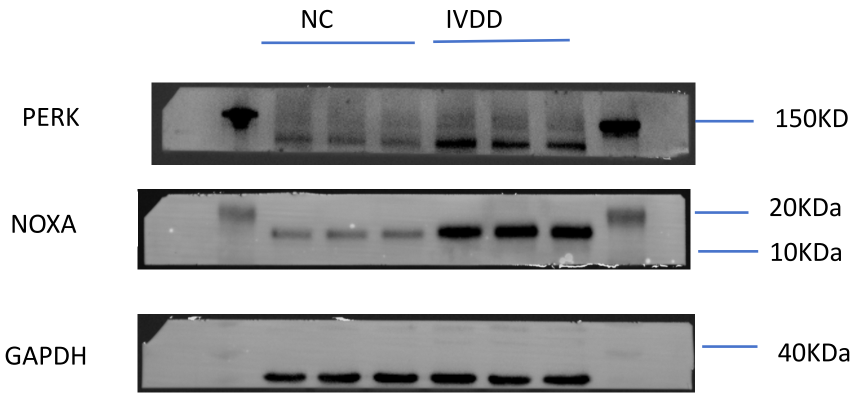

Uncropped gels for Western Blots in Figure 2

Figure 2-A

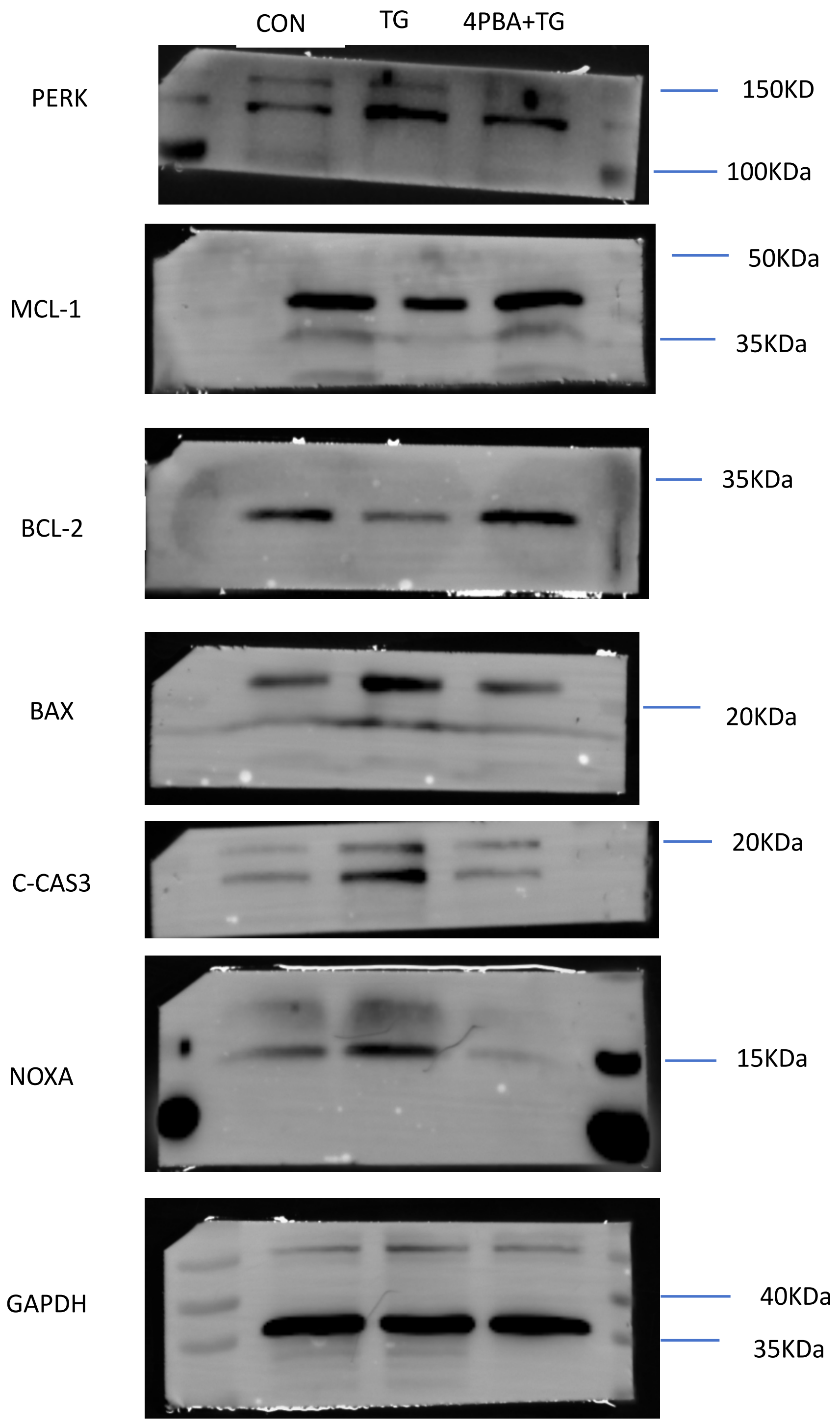

Figure 2-G

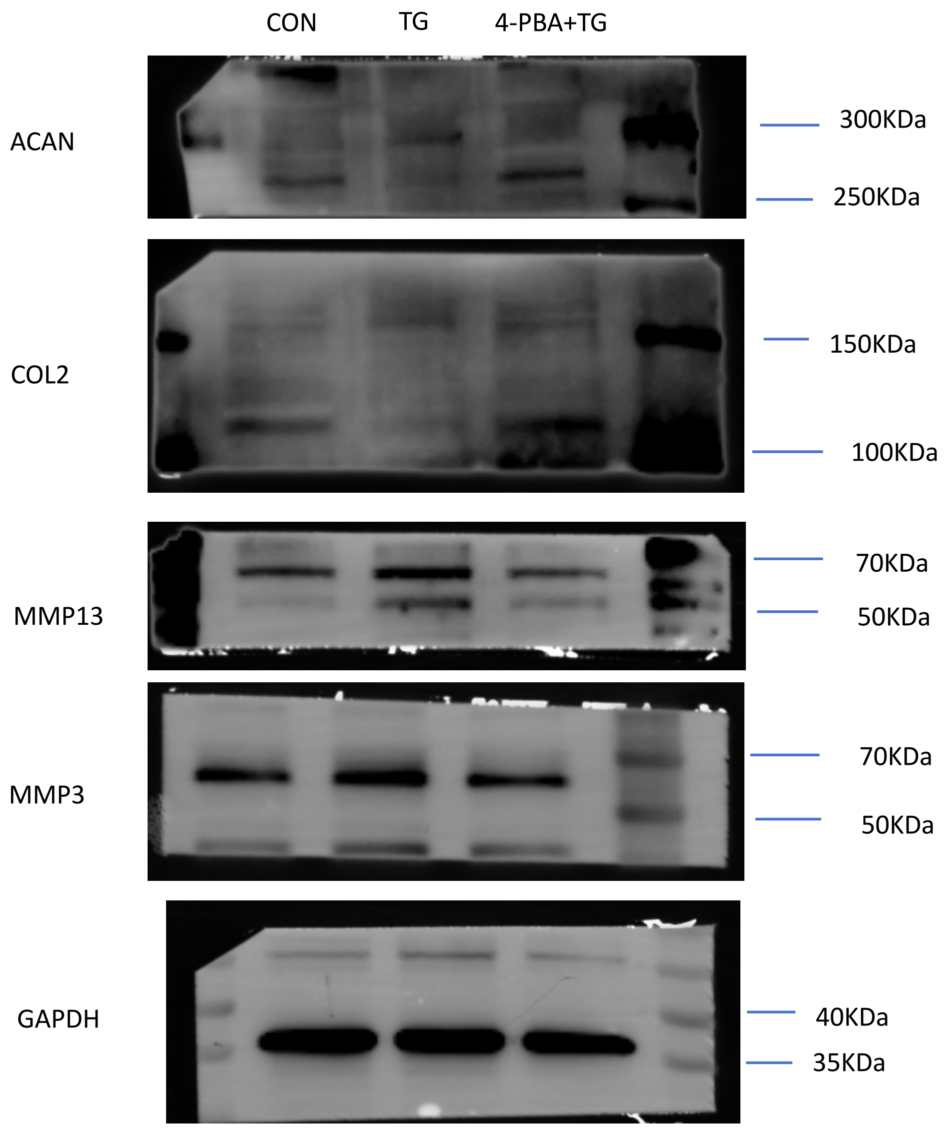
CON
TG
4-PBA+TG
ACAN
300KDa
250KDa
COL2
150KDa
100KDa
MMP13
70KDa
50KDa
MMP3
70KDa
50KDa
GAPDH
40KDa
35KDa

Uncropped gels for Western Blots in Figure 3

Figure 3-A

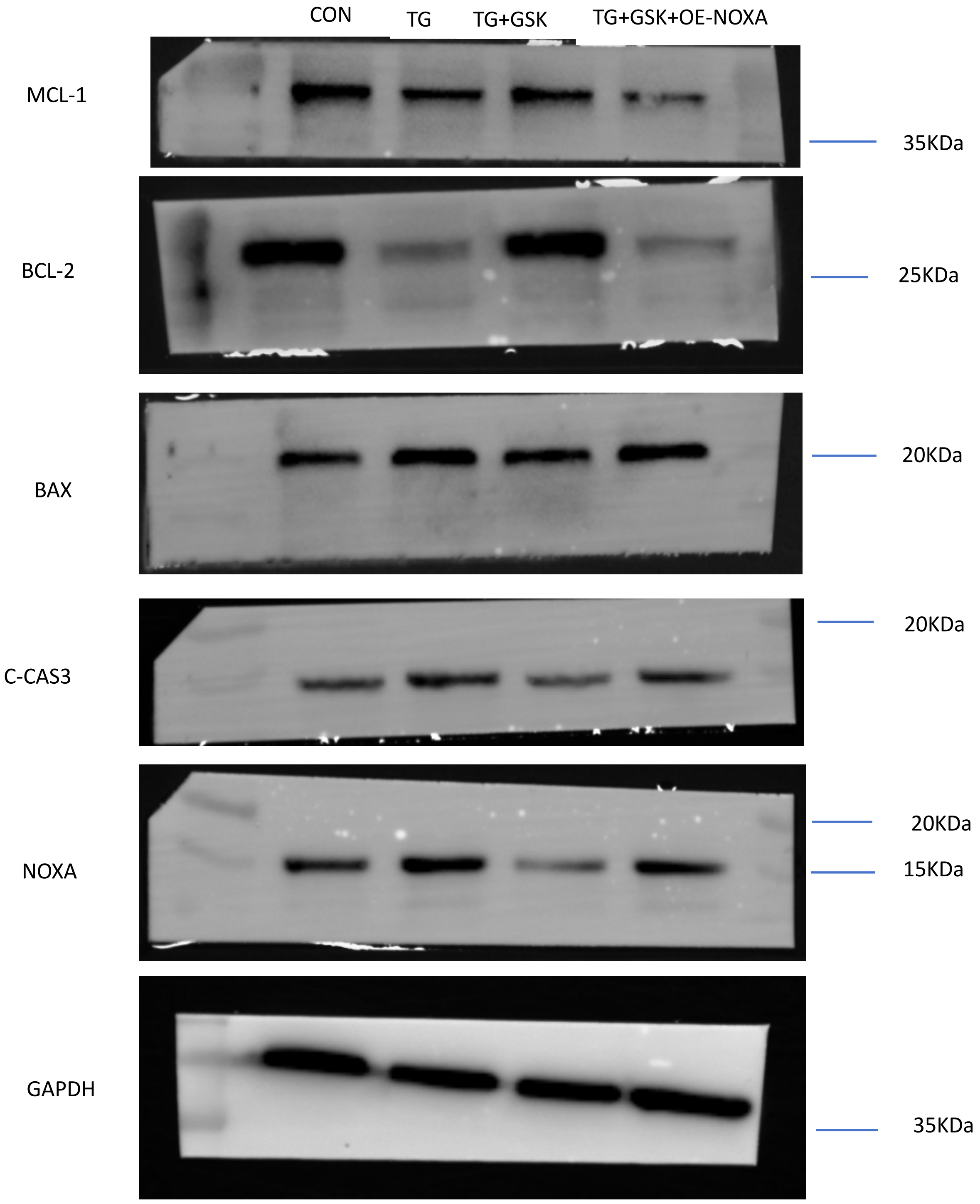

Figure 3-I

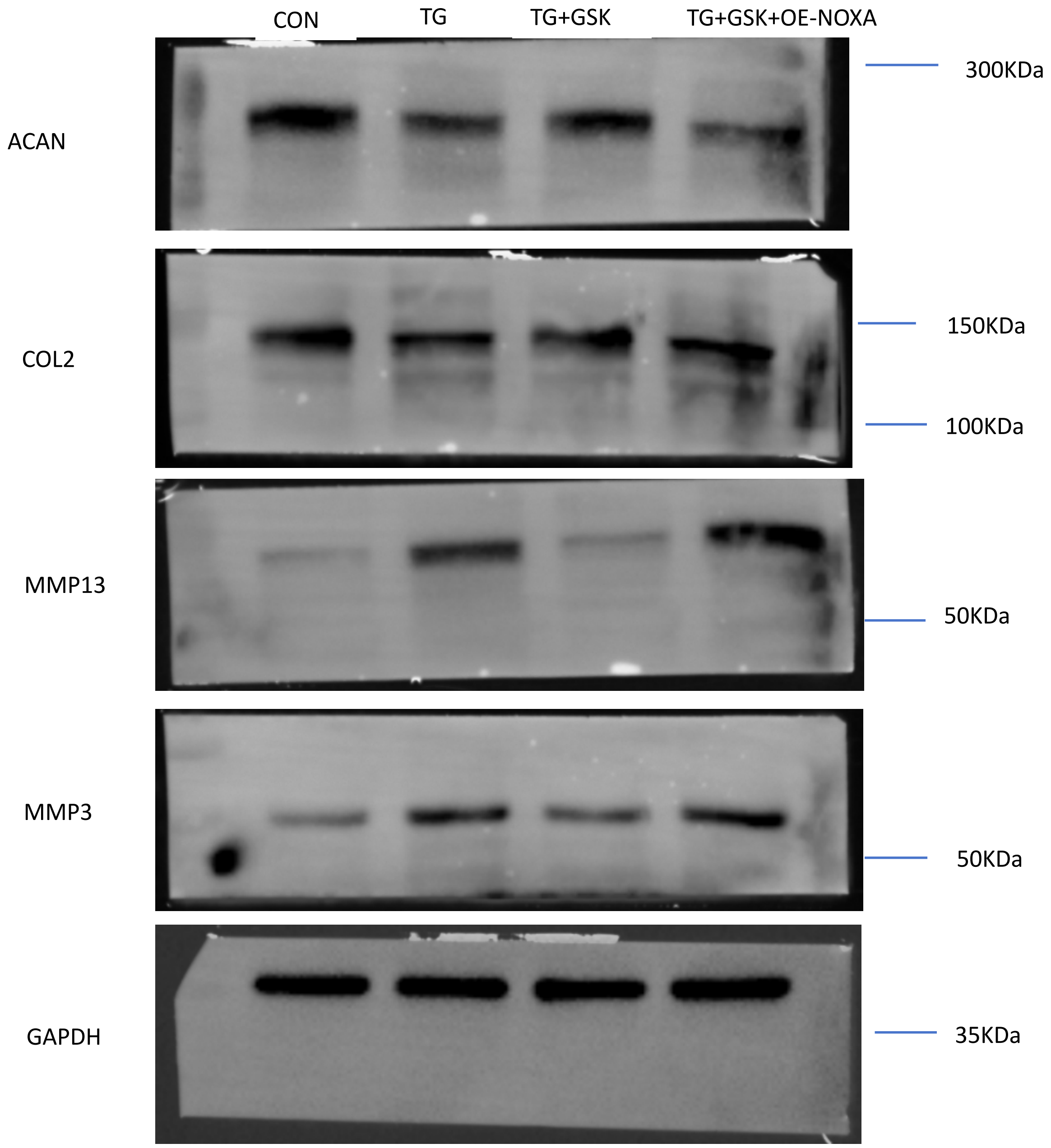
CON
TG
TG+GSK
TG+GSK+OE-NOXA
ACAN
300KDa
COL2
150KDa
100KDa
MMP13
50KDa
MMP3
50KDa
GAPDH
35KDa

Uncropped gels for Western Blots in Figure 4

Figure 4-A

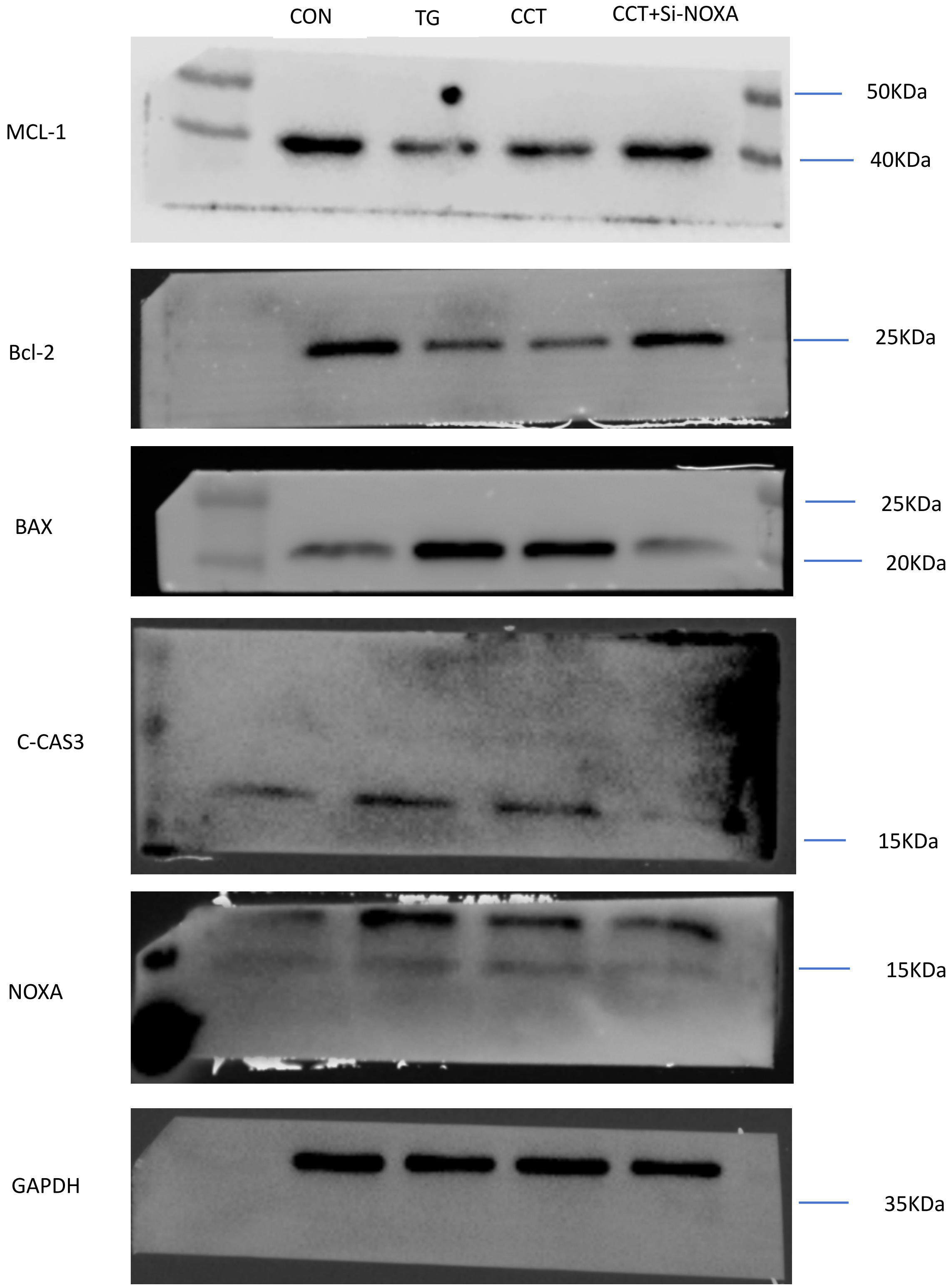

Figure 4-K

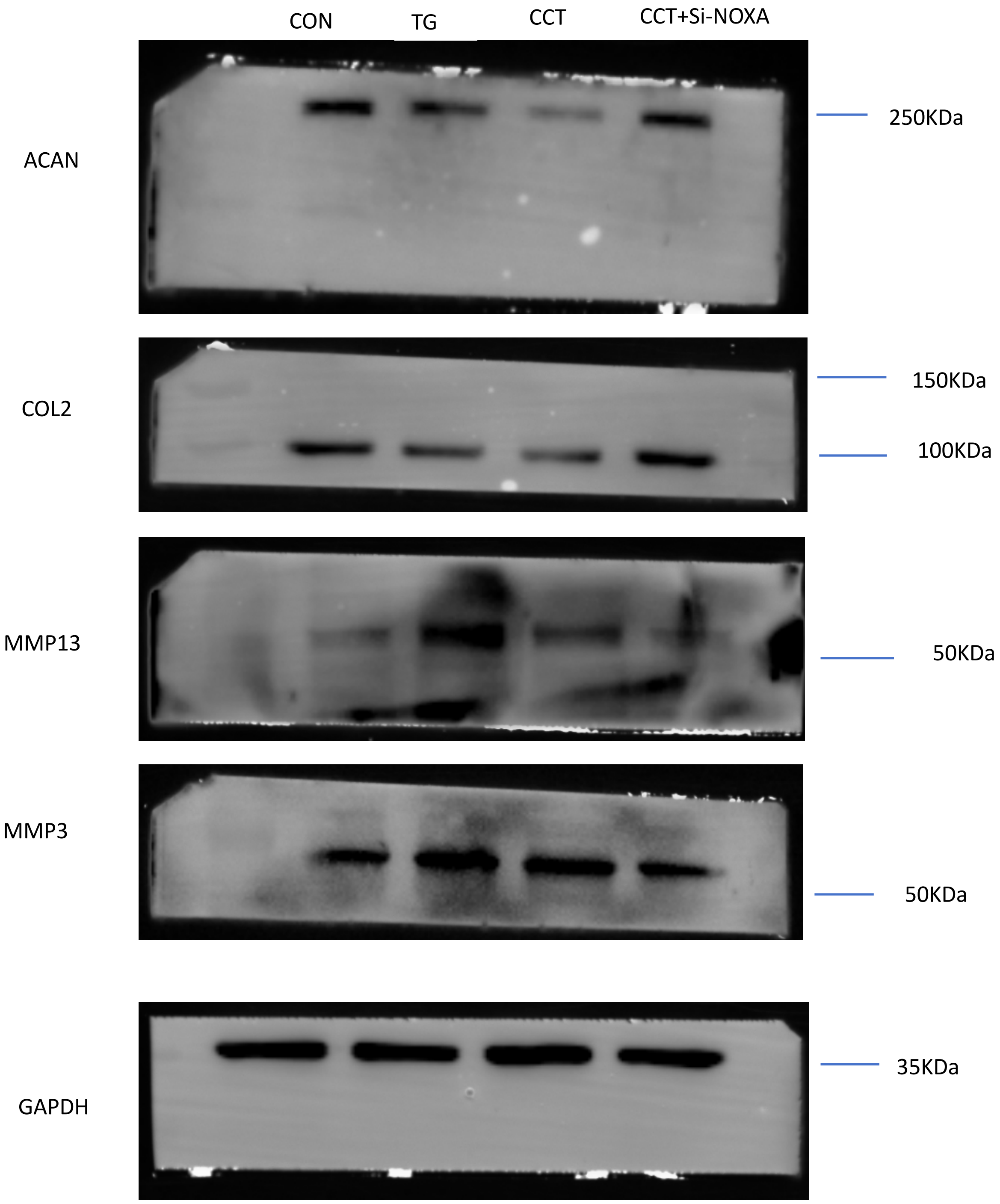
CON
TG
CCT
CCT+Si-NOXA
ACAN
250KDa
COL2
150KDa
100KDa
MMP13
50KDa
MMP3
50KDa
GAPDH
35KDa

Uncropped gels for Western Blots in Figure S3

Figure S3A

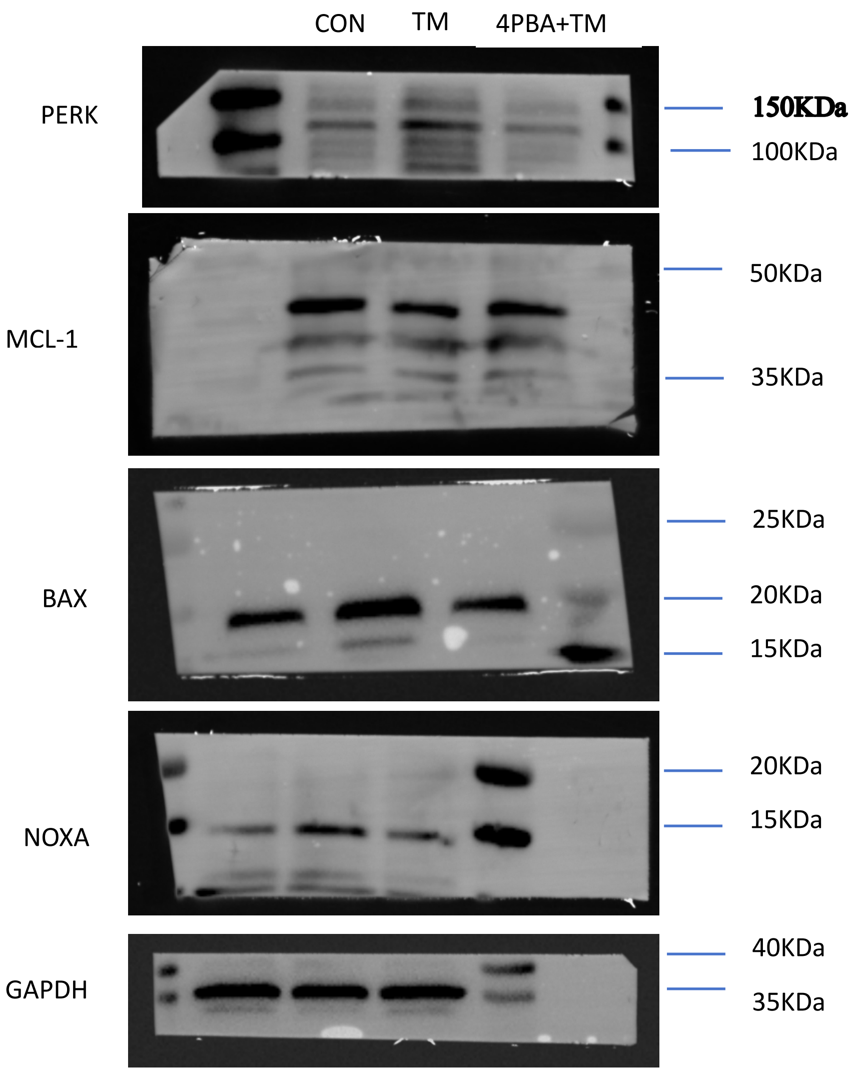

Uncropped gels for Western Blots in Figure S5

Figure S5-C

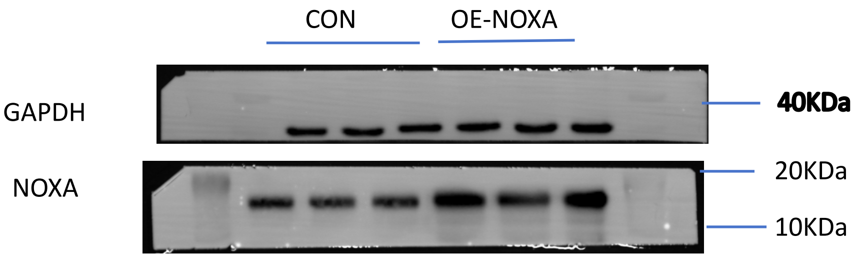


Figure S4-G

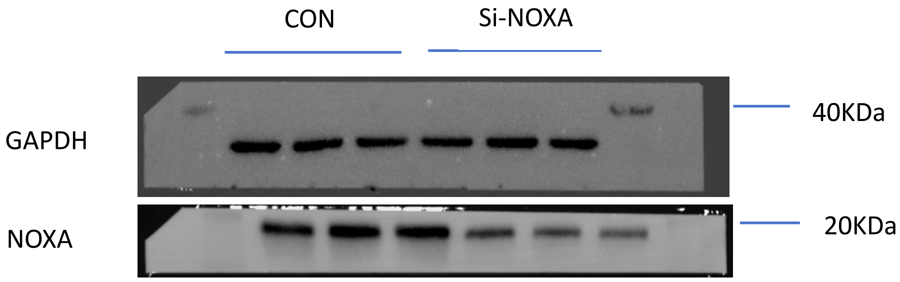

Supplement: Supplementary file 2 — Uncropped gels image for Western Blot [file 41420_2025_2539_MOESM2_ESM.pdf]
